# Association between early life second-hand smoke exposure on child sleep and psychoactive substance use on adult sleep patterns in an urban informal settlement in Uganda

**DOI:** 10.1371/journal.pone.0312127

**Published:** 2025-01-03

**Authors:** Solomon T. Wafula, Lydia N. Namakula, John B. Isunju, Richard K. Mugambe, Tonny Ssekamatte, David Musoke, Rhoda K. Wanyenze

**Affiliations:** 1 Department of Disease Control and Environmental Health, School of Public Health, Makerere University, Kampala, Uganda; 2 Department of Infectious Disease Epidemiology, Bernhard Nocht Institute for Tropical Medicine, Hamburg, Germany; Makerere University, UGANDA

## Abstract

**Background:**

Psychoactive substance use in adults and second-hand smoke (SHS) exposure among children are leading contributors to sleeping problems. Despite this, there is limited data on how these exposures influence sleep patterns in informal settings. Our study assessed the associations between substance use, SHS exposure and sleep disturbances among adults and children in an urban informal settlement in Uganda.

**Methods:**

We conducted a cross-sectional study in an urban informal settlement in Kampala, Uganda. Data was collected on self-reported sleep problems among adults including sleep duration, insomnia and sleep dissatisfaction, as well, as sleep-disordered breathing (SDB) in children. We evaluated children’s early-life SHS exposure and psychoactive substance use in adults using questionnaires. We modelled the associations between the exposures and sleep problems in adults and children using modified Poisson regression.

**Results:**

Data were collected from 284 adults, who also reported on their children’s sleep experience. Among adults, 59.2% reported insufficient sleep (less than 7 hours), 34.9% experienced insomnia, and 28.3% were dissatisfied with their sleep patterns. Active smoking was associated with insomnia (Prevalence Ratio (PR) = 2.74, 95% Confidence Interval (CI): 1.14–6.59), and alcohol use was associated with sleep dissatisfaction (PR = 1.81, 95% CI 1.23–2.69). In children, 40.0% (88/220) exhibited SDB problems. Those exposed to SHS either during pregnancy or within six months post-birth had a higher risk of SDB than unexposed children (PR = 1.78, 95% CI 1.21–2.61). The risk was also elevated for children exposed to SHS during both periods (PR = 1.48, 95% CI 1.02–2.13).

**Conclusions:**

Our findings suggest that smoking was associated with insomnia and alcohol with sleep dissatisfaction among adults. Early-life SHS exposure was associated with an increased risk of SDB in children. These results emphasize the need to support ongoing public health initiatives and maintain a smoke-free environment, particularly for children in their early life.

## Introduction

Sleep disorders are a growing public health concern globally, affecting both adults and children. Substance use is linked with sleep problems. Evidence indicates that nearly 70% of patients admitted for detoxification report sleep problems before admission, and 80% of those who report sleep problems attribute them to psychoactive substance use [1]. Sleep disorders affect at least 1 in 4 adults but prevalence can be as high as 60% in certain populations and settings [2, 3]. Sleep problems can increase the risk of chronic conditions such as mental health disorders, hypertension, and cardiovascular diseases [4, 5]. Residents of informal urban settings have increased vulnerabilities to substance use and sleep problems [6–10]. In such informal settings, there are overcrowded living conditions, excessive noise environments, and exposure to various environmental pollutants, all of which can disrupt sleep [10]. In Uganda, sleep-related problems have not been thoroughly explored especially how substance use or exposure therefore of affects sleep quality. While some studies on sleep problems in the general population have been conducted, very few have focused on residents of informal settlements [11]. This gap in knowledge limits our understanding of the unique challenges faced by this demographic and their specific sleep-related needs, which impedes the creation of targeted interventions to improve their sleep and overall health outcomes.

Optimal sleep quality is also crucial for the health and well-being of young children. While these children are not typically substance users, they remain vulnerable to second-hand smoke (SHS) from tobacco or marijuana, which is harmful to their health [12–15]. SHS exposure can disrupt the sleep cycle and make it difficult for children to fall asleep and stay asleep [16–19]. This has been reported to be associated with cognitive impairments such as reduced perception, memory and mood [15, 20–23].

The relationship between psychoactive substance use /exposure and sleep difficulties is complex and bidirectional. Insufficient sleep can also lead individuals to self-medicate with psychoactive substances which can lead to drug abuse/addiction.

While the associations between the use of psychoactive substances (among adults) and exposure to SHS among children and sleep disturbances have been documented in developed countries [24, 25], such studies in sub-Saharan Africa (SSA), particularly in Uganda are scarce. Studies have often not controlled for important confounders such as mental health and indoor air quality [20, 26]. This study aimed to fill this gap by assessing the association between psychoactive substance use or SHS exposure, and self-reported sleep problems among adults and children in an informal setting in Kampala, Uganda.

## Methods

### Study design and setting

This was a cross-sectional study in design and employed quantitative data collection methods. Between April and May 2022, the study was carried out in Bwaise, an informal settlement in Kampala City, Uganda [27], Uganda. The study was restricted to three densely populated zones: Bwaise I, II and III. The zones of Bwaise I, Bwaise II, and Bwaise III have average populations of approximately 37,500, 42,000, and 35,000 people, respectively [27]. In Kampala’s informal urban settings, it’s estimated that over 50% of adults use psychoactive substances [28].

### Study population and inclusion criteria

The study population consisted of both adults (aged 18 years or more) and all children aged 6–59 months residing in the selected households. The selected adult (one per household) provided information about themselves and their household. Additionally, adults were requested to provide demographic and sleep disorders data for any child aged 6 to 59 months in their household (if they existed). Inclusion criteria included living in selected households for at least 6 months and providing informed consent. Exclusion criteria included residence of less than 6 months in the area, having cognitive impairments or severe aggression, not being available after two visits to the household on the same day or not consenting.

### Sample size and sampling

**Fig 1:**
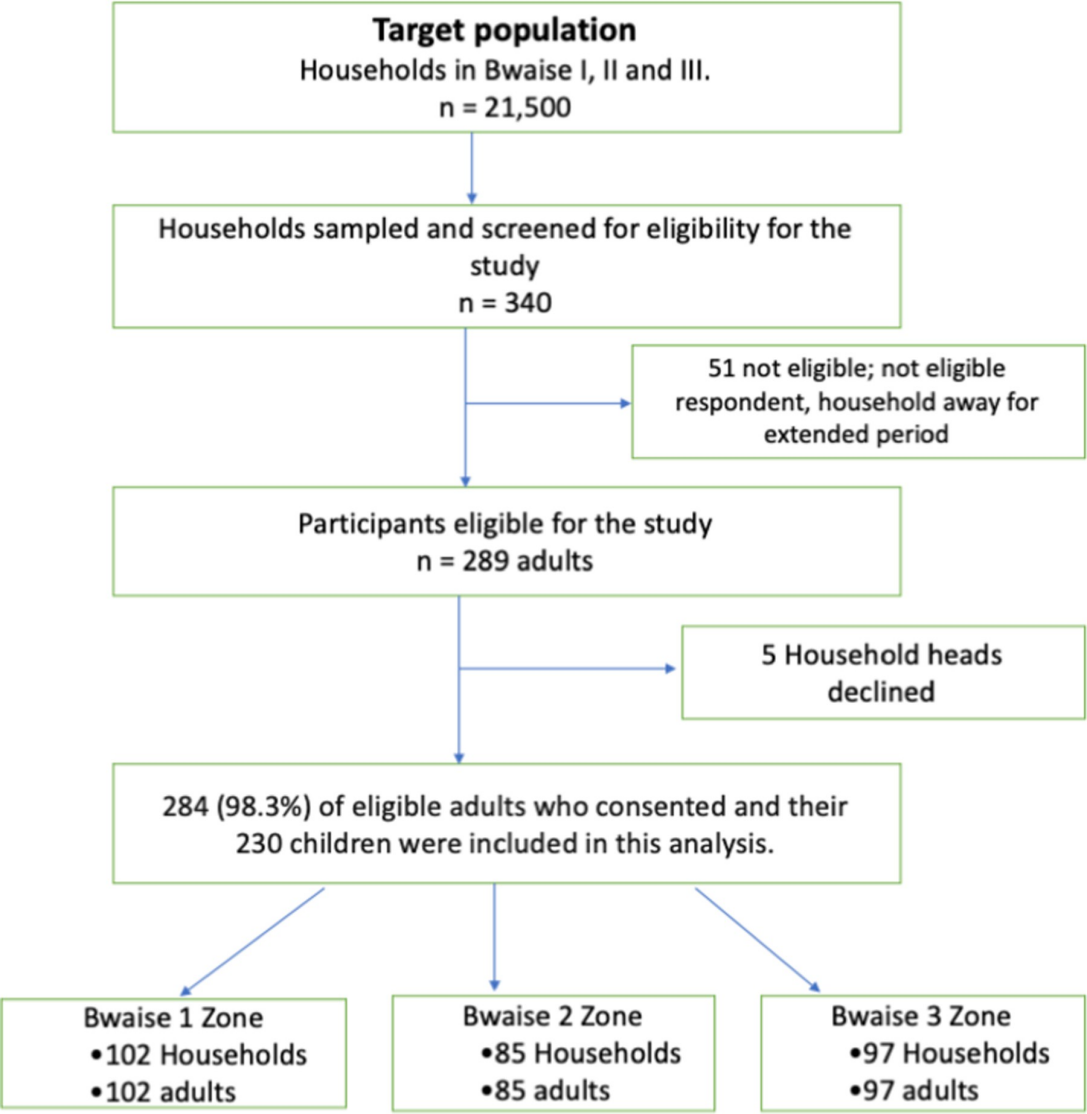
Flow chart for sample selection.

We calculated a sample size of 300 participants using the Kish Leslie formula [29]. We assumed the prevalence of sleep problems (severe sleep difficulty) of 23.0% from a previous study in Uganda [30]. We also considered 80% power, 95% confidence intervals, 5% margin of sampling error and 5% to cater for potential non-response. Therefore, we estimated 300 adults and 300 children. We randomly selected three parishes from Bwaise and one village from each parish using the ballot method. Then, we systematically sampled the number of households from each village (proportionate to size), using a sampling interval determined by the ratio of the number of households in the village and the desired sample size from that village. We selected three villages that were close to each other and had similar characteristics including population size (homogenous). We did not expect any cluster effect. We interviewed one eligible adult person per household if they consented. Workflow is shown in **Fig 1.**

### Data collection

Experts reviewed the literature and developed questionnaires with questions added based on face and content validity. Before actual interviews, we trained research assistants on data collection tools, research ethics and quality control procedures, then pretested the tools with 15 participants in a similar informal urban settlement in Kampala to check if questions were clearly understood by respondents, after which modifications were made to address any ambiguities. Data were collected using a structured interviewer-administered questionnaire on the KoboCollect app installed on hand-held Android devices. The digital tools were fitted with necessary filters and branching logic to record valid responses. For adults, we collected data on sociodemographic characteristics, use of psychoactive substances, sleep problems and mental health (depression and anxiety). Indoor air conditions were measured for PM2.5, PM10 and CO over five hours (twice per hour from 9 am to 2 pm). Ten three-minute average readings were taken in the living room, with sensors placed on a table one meter above the ground and at least 1.5 meters from windows and door. We also obtained a self-report on whether spraying for insecticides indoors happens sometimes or often. Parents or guardians provided data on their children’s age, gender, sleep-disordered breathing, and early life exposure to SHS.

### Measurement of variables

#### Outcome measurement

For adults, we assessed three outcomes: insufficient sleep, insomnia and sleep dissatisfaction. We defined insufficient sleep as sleeping less than 7 hours per day on average on most days, based on the consensus statement recommendation [31]. We defined insomnia as having difficulty falling asleep and staying asleep at least three times per week, despite having enough opportunity to sleep, as per the American Psychiatric Association [32] To measure insomnia, we asked participants two questions about their sleep patterns in the past 30 days. The first question was: “Did you have trouble sleeping, even when you had enough time to sleep?” The second question, for those who said yes to the first one, was: “How often did you have trouble sleeping?” We coded insomnia as having trouble sleeping at least two times a week. Lastly, we assessed self-reported sleep dissatisfaction in the past 30 days of the study (binary variable). We asked them a single question: “Are you satisfied or not with your sleep patterns and durations in the past 30 days?” and coded yes, satisfied as 1 and no, not satisfied as 0. A team of five experts checked the face validity of this outcome and found it acceptable.

For children, we assessed sleep-disordered breathing problems (SDB), which are marked by nighttime gasping, snoring, or difficulty breathing. We measured SDB using the SDB domain of the validated “Sleep Disturbance Scale for Children (SDSC)” tool [33]. We coded SDB as present (1) if the frequency was “sometimes/almost always/most of the time” and absent (0) if the frequency was “Never/seldom”.

#### Exposure measurement

Among adults, the exposure was defined as using any of these substances in the past 30 days: alcohol, tobacco or marijuana. Each substance was a separate exposure. The non-exposure to a substance was defined as not using it, regardless of other substances. The exposure status for each substance was determined by the participants’ self-report.

We also interviewed parents about their children’s exposure to secondhand smoke (SHS). We considered a child exposed to SHS if the parents answered yes to either of these questions: Did anyone smoke while the child’s mother was pregnant and living in the same house? Did anyone smoke inside the house where the child lived for the first six months of life? We categorized the children’s early-life SHS exposure into three groups: (1) unexposed (no SHS exposure during pregnancy or first six months of life); partially exposed (SHS exposure during pregnancy or first six months of life, but not both); and fully exposed (SHS exposure during both pregnancy and first six months of life) [20].

#### Covariates

We chose covariates based on the Directed Acyclic Graph [34]. These covariates were 1) Sociodemographic characteristics: parental education (“no formal education”, “primary”, and “post-primary education”), marital status (married, singled, widowed/separated), children under 5 (“yes”, “no”), 2) Current depression symptoms (PHQ-9 score of 10 or higher) [35] and generalized anxiety disorder (GAD-7 scale score of 10 or higher) [36], 3) Respiratory problems (in children): phlegm (“yes”, “no”), and wheezing(“yes”, “no”), 4) Adults self-reported health status (“fair/poor” coded 1, “Excellent/very good/good” coded 0), 5) Indoor dampness and air pollution: Dampness, particulate matter PM2.5 and PM10 and carbon monoxide as measured in our previous study [37]. High PM2.5, PM10 or Carbon monoxide were defined as pollutant levels above the median levels of the study households. Low levels were defined as pollutant levels below or equal to the median levels. Measurements of these pollutants were described in an earlier publication [37] 6) Hypertension. We used the American Heart Association guidelines to define hypertension as a blood pressure measurement of SBP 140 or/and DBP 90 mm Hg. Normotension was defined as a blood pressure measurement of less than SBP 140 and DBP 90 mm Hg. Classifications were based on the average of three readings taken in a relaxed position within three minutes on the same day.

### Statistical analysis

We used the Kobo Collect app to collect data and exported it as a comma-separated (CSV) file to Stata 14 (Stata Corp, Texas, USA) for cleaning and analysis. We used frequencies, percentages, median and interquartile ranges to describe the categorical and numerical data. We used a generalized linear model of the poison family with a logarithmic link function and robust error variance to examine the association between psychoactive substance use or exposure and sleeping problems in adults and children [38]. We chose robust Poisson regression over logistic regression because logistic regression can overestimate the effect size when the outcome is common (i.e. p > 10%) [39]. We included all relevant covariates identified apriori (through DAG theory) in the multivariable model to reduce confounding bias. We reported the adjusted prevalence ratios and their 95% confidence intervals. We used a complete case analysis approach because the missing data was very low (<3%).

### Ethics considerations

Ethical approval was obtained from Makerere University School of Public Health Higher Degrees, Research and Ethics Committee (Ref No. SPH-2021-99) and Uganda National Council for Science and Technology (UNCST)-Ref No. SS996ES. Before conducting the study, administrative clearance was obtained from Kampala Capital Authority [27] Department of Health and Environment. All participants were interviewed after informed consent and in strict adherence to the approved risk mitigation protocols for COVID-19 prevention. Privacy and confidentiality were ensured throughout the study.

## Results

### Characteristics of respondents

Complete data were available from a total of 284 adults (response rate = 94.6%) and 230 children Among 284 adults, most were female (85.2%) and tenants (90.8%). Half were under the age of 30 years (51.4%), had post-primary education (51.1%) and had only lived in the area for less than five years (52.1%).

Among the children, 52.6% (121/230) were females, and 72.2% (166/230) were not yet in school. The median age of children was 24 months (IQR = 4,48). Approximately 36.0% (67/214) of the children had been exposed to SHS during pregnancy, while 40.4% (88/218) were exposed to SHS in the first six months (household member who smoked) and 31.3% (67/214) had been exposed both prenatally and in the first 6 months of their life and 13.6% (29/214) were exposed to SHS either during pregnancy or in first 6 months (but not both periods) (Table 1).

**Table 1:** Sociodemographic characteristics of household participants.

Characteristics of adults	Number of participants (N = 284)	Percent (%)
**Gender:** Female	242	85.2
**Age of respondents (in years)**		
< 30	146	51.4
30–45	108	38.0
> 45	30	10.6
**Marital status**		
Married or living with a partner	136	47.9
Separated	43	15.1
Single	105	37.0
**Education level**		
No formal education	25	8.8
Primary	114	40.1
Post-primary	145	51.1
**Occupation**		
Business	137	48.2
Formal or informal employment	58	20.4
Unemployed	72	25.4
Other	17	6.0
**Owner of the dwelling**		
Yes (Owner)	26	9.2
No (Tenant)	258	90.8
**Household monthly income (USD)**		
< 50	77	27.1
50–150	163	57.4
> 150	44	15.5
**Duration of stay in the informal settlement (in years)**		
< 5	148	52.1
5–10	57	20.1
> 10	79	27.8
Smoking in the previous 30 days: Yes	31	14.4
Marijuana use in the previous 30 days	19	6.7
Alcohol use in the previous 30 days	109	38.4
**Mental Health**		
Anxiety	38	13.4
Depression	96	33.8
**Child characteristics (n = 230)**		
**Gender: Female**	121	52.6
**Education status of the child**		
Not yet in school	166	72.2
Schooling (nursery)	64	27.8
**Exposure to SHS in early life**		
No exposure	118	54.4
Ever exposed during pregnancy or the first 6 months of life	29	13.6
Exposed during both pregnancy and the first six months of life	67	31.3

### Sleeping problems in adults and children

The median sleep duration for adults on a typical working day was 6hrs (IQR = 4,8) and 7hrs (IQR = 4,9) on non-working days. Nearly 6 in 10 adults (59.2%) reported sleeping on average less than 7 hours on working days and hence considered to have insufficient sleep. In contrast, 34.9% of adults had insomnia symptoms, and 28.3% were dissatisfied with their sleep patterns and amounts. In children, it was reported that 40.0% sometimes or always snored, gasped or faced difficulties in breathing at night hence classified as having sleep-disordered breathing problems (Table 2).

**Table 2:** Sleep patterns and disorders in adults and children.

Attributes	Summary statistic
**In adults (N = 284)**	
Sleep duration on a typical working day (in hours): median (IQR)	6 (4–8)
Sleep duration on the non-working day (in hours): median (IQR)	7 (4–9)
**Insufficient sleep (less than 7 hours) on a working day (n = 168)**	59.2%
Trouble falling asleep and/or staying asleep in the last 14 days (n = 99)	34.9%
Dissatisfied with own sleep patterns and amounts (n = 82)	28.29%
**In children (N = 230)**	
**Sleep-disordered breathing problem (snoring, gasping, difficulties in breathing at night)**	
Almost always (n = 14)	6.4%
Sometimes (n = 74)	33.6%
Seldom or never (n = 132)	60.0%

### Associations between psychoactive substances and sleeping problems among adults

Participants who were regular smokers were more likely to report having difficulties falling asleep or staying asleep (PR = 2.74, 95% CI 1.14–6.59), while alcohol use (PR = 1.81, 95% CI 1.23–2.69) was associated with reporting sleep dissatisfaction.

Among other predictors, adults who had separated from their spouses were 47% more likely to report insufficient sleep (PR = 1.47, 95% CI 1.16–1.87). Higher indoor carbon monoxide levels were associated with a higher incidence of insufficient sleep (PR = 1.38, 95% CI 1.14–1.68). Those who had children under five in their care were also more likely to report insufficient sleep (PR = 1.26, 95% CI 1.04–1.53) (Table 3). The distribution of sleep outcomes by attributes is available as supplementary file **S1 Table.**

**Table 3:** Associations between psychoactive substances and sleeping problems in adults (adjusted regression).

Attributes	Insufficient sleep PR (95% CI)	Insomnia symptoms PR (95% CI)	Sleep dissatisfaction PR (95% CI)
**Gender:** Male	1.22 (0.97–1.52)	0.96 (0.57–1.60)	1.22 (0.80–1.87)
**Age (in years)**			
**< 30**	1	1	1
**30–45**	1.07 (0.87–1.32)	1.09 (0.78–1.51)	0.67 (0.45–0.99)
**> 45**	1.02 (0.76–1.38)	0.76 (0.42–1.40)	0.93 (0.53–1.63)
**Marital status**			
Married	**1**	1	1
Separated	**1.47 (1.16–1.87)**		
Single	1.11 (0.90–1.38)		
**Occupation**			
Business			1
Employed			0.69 (0.43–1.12)
Unemployed			0.68 (0.41–1.13)
Other			0.30 (0.08–1.10)
**Household income**			
< 50		1	1
50–150		1.25 (0.82–1.91)	1.42 (0.87–2.33)
>150		1.65 (1.01–2.68)	0.86 (0.39–1.90)
**High PM2.5 levels**	0.72 (0.60–0.87)		
**Carbon monoxide levels: high**	**1.38 (1.14–1.68)**		
**Current smoking**		**2.74 (1.14–6.59)**	1.04 (0.66–1.63)
**Marijuana use**			1.57 (0.95–2.62)
**Alcohol use**		0.75 (0.52–1.07)	**1.81(1.23–2.69)**
**Indoor spraying for insecticides**		0.74 (0.52–1.07)	
**Hypertension**			1.20 (0.83–1.74)
**Have children under 5**	**1.26 (1.04–1.53)**		
**Self-reported poor health**			1.31 (0.92–1.86)

### Association between psychoactive substances (including SHS exposure) and sleep-disordered breathing problems in children

In the adjusted analysis, we found that either prenatal exposure or exposure to SHS in the first 6 months of life (PR = 1.78, 95% CI 1.21–2.61) was associated with sleep-disordered breathing (SDB) problems among children (6–59 months). Where there was exposure both prenatally and in the first 6 months, a significant effect on SDB was also observed (PR = 1.48, 95% CI 1.02–2.13) compared to those with no exposure. Additionally, children from households with higher incomes i.e., > USD 150 (PR = 0.25, 95% CI 0.10–0.64) were less likely to report SDB compared to those who earned < 50 USD. Child wheezing (PR = 1.60, 95% CI 1.17–2.18) and phlegm (PR = 1.51, 95% CI 1.021–2.23) were also associated with reports of SDB (Table 4).

**Table 4:** Predictors of sleeping-disordered breathing problems in children.

Attributes	Sleep-disordered breathing problem		Adjusted PR (95% CI)
	No (132)	Yes (88)	
**Sex of the child**			
**F**emale	65 (55.1)	53 (44.9)	1
Male	67 (65.7)	35 (34.3)	0.91 (0.66–1.24)
**Age of the child (months)**			1.00 (1.00–1.01)
**Marital status of the guardian**			
Married	78 (66.7)	39 (33.3)	1
Separated	15 (50.0)	15 (50.0)	1.11 (0.71–1.73)
Single	39 (53.4)	34 (46.6)	1.03 (0.73–1.43)
**Education status of the child**			
Not yet in school	101 (64.3)	56 (35.7)	1
In school	31 (49.2)	32 (50.8)	1.26 (0.92–1.73)
**Household income (USD)**			
**< 50**	28 (42.4)	38 (57.6)	1
**50–150**	73 (61.9)	45 (38.1)	**0.67 (0.49–0.93)**
**>150**	31 (86.1)	5 (13.9)	**0.25 (0.10–0.64)**
**Marijuana use by a household member**			
No	119 (58.3)	85 (41.7)	
**Y**es	13 (81.3)	3 (18.8)	0.58 (0.24–1.44)
**Indoor spraying for insecticides**			
No	97 (67.4)	47 (32.6)	
Yes	35 (46.0)	41 (54.0)	1.28 (0.93–1.75)
**Respiratory problems**			
**Phlegm**			
No	121 (64.0)	68 (36.0)	1
Yes	11 (35.5)	20 (64.5)	**1.51 (1.03–2.23)**
**Wheezing**			
No	115 (66.1)	59 (33.9)	**1**
Yes	17 (37.0)	29 (63.0)	**1.60 (1.17–2.18)**
**Early life exposure to SHS**			
No exposure	84 (71.2)	34 (28.8)	1
Either during pregnancy or in the first 6 months	10 (35.7)	18 (64.3)	1.78 (1.21–2.61)
Exposed during both pregnancy and the first six months of life	33 (49.3)	34 (50.8)	**1.48 (1.02–2.13)**

## Discussion

This study aimed to assess the association between psychoactive substance use, early life exposure to SHS and self-reported sleeping problems among adults and children in an informal setting in Kampala, Uganda. The findings reinforce previous research about the detrimental effects of psychoactive substances on exposed individuals’ sleep quality in adults as well as children and require the urgent need to address the modifiable risk factors [20, 29, 40].

### Sleep problems and psychoactive substance use in adults

In our study, we found that active smoking is associated with insomnia symptoms which is consistent with earlier research conducted in various populations and urban informal settings [41–43]. Specifically, previous studies conducted in more affluent settings have reported clear associations between nighttime smoking, greater insomnia, and shorter sleep [44, 45]. Nicotine, an active ingredient in cigarettes and tobacco, acts as a stimulant that increases alertness; and hence higher risk of insomnia [46]. The nicotine effect not only stimulates the craving for more tobacco but also prompts individuals to smoke during the night and early morning hours to alleviate cravings and counteract symptoms such as increased alertness, anxiety, and restlessness [44, 46, 47]. The stuffiness and poor air circulation around most informal dwellings would increase the effect of nicotine if the smoking happens indoors [48]. Our findings have important implications for public health in Uganda, as smoking rates, particularly among men have increased [49]. This suggests that smoking cessation interventions may be an important strategy for improving sleep outcomes in Uganda. Furthermore, given the known negative health consequences of smoking, such as increased risk of cancer, heart disease, and stroke, addressing smoking as a risk factor for sleep problems may have additional benefits beyond improving sleep quality.

Several researchers have found associations between the use of substances such as cannabis, khat and alcohol and sleep apnea and overall poor-quality sleep [17, 50–52]. Our findings that alcohol use is associated with increased risks of sleep dissatisfaction align with this body of research [53, 54]. Whereas, the literature suggests that quantity and type of alcohol or drug consumed before sleep is a determining factor with specific types of alcohol—beyond beer and wine—being implicated in poorer sleep outcomes [55, 56], we did not investigate this in our study, and this warrants further investigations to understand associations between alcohol type and quantity and sleep quality among informal settlement residents. The study provides a unique insight into the sleep patterns among informal settlement residents in Uganda as very few studies have investigated this. The findings therefore suggest that interventions aimed at minimizing alcohol and marijuana use may be useful in reducing sleep dissatisfaction among residents of informal settlements in Uganda.

### SHS and sleep problems in children

Regarding second-hand smoke (SHS) and its impact on children’s sleep health, we found that early-life SHS exposure was associated with greater sleep problems in children. Specifically, the risk of sleep-disordered breathing (SDB) is higher in children with either prenatal or early postnatal SHS exposure or both. Our findings add to the growing body of research highlighting that SHS exposure results in significant increases in SDB among children exposed [20, 26, 57, 58]. High rates of active smoking and prolonged SHS exposure in informal urban settings pose significant risks, particularly as nicotine adversely affects fetal lung development and airway structure [59–61]. Similar findings have been identified among non-smoking adults with SHS exposure [62]. These results are important for public health interventions aimed at reducing SHS exposure among children in these informal settings.

One of the mechanisms through which SHS exposure may affect children’s sleep has been suggested as an exacerbation of respiratory symptoms which in in turn, can lead to sleep disturbances or restlessness [26]. This study found that children with respiratory problems such as phlegm and wheezing were 48% and 59% more likely to report SDB, respectively. Sleep and recumbency naturally impair gas exchange and reduce oxygen stores which is further aggravated in individuals with respiratory ailments leading to an increased risk of SDB [63]. Different studies have found an increased likelihood of wheezing and asthma among children exposed to maternal smoking and a significant association between SDB and respiratory conditions [64–66]. Our findings suggest that respiratory complications could play a mediating role in the relationship between SHS exposure and sleep disturbances, warranting further investigations in large longitudinal studies. Implementing simple ventilation strategies, such as creating small openings near the roofline or using low-cost mesh screens on windows, can significantly enhance air quality and oxygen levels within homes. The cost-effective measures can alleviate the discomfort caused by stuffiness, potentially improving sleep quality for children [67]. However, it is important to note that there are no known /clear mechanisms for the effect of SHS exposure on the sleep of children hence the need for further research [26].

### Limitations and strengths

Several factors need to be considered as limitations in this study. The first is that small size and non-response may limit the power of the study, rendering the association effects less robust enough (largely speculative). Secondly, the cross-sectional nature of data cannot allow us to infer causality; hence, longitudinal studies are needed to demonstrate causal inference. Thirdly, self-reported measures of SHS exposure introduce the potential for recall bias, leading to potential non-differential misclassification of the exposures. Fourth, while we observed a consistent association between SHS (whether SHS exposure happened either during the prenatal period or in the first 6 months or both periods) and SDB in children, future studies should employ more objective measures of SHS for precise estimates. However, this study is one of the first to contribute evidence on the associations between substance use in adults and early-life SHS exposure in children on sleep outcomes, especially in urban poor settings in Uganda.

## Conclusion

Among adults, smoking and alcohol were associated with trouble sleeping and sleep dissatisfaction, respectively. Among children, exposure to early-life SHS increased the odds of sleep-disordered breathing problems. Problems of poor sleep should not be ignored especially in children due to its effects on behaviour, and mental and cognitive problems during their growth. Therefore, the findings underscore the need for deliberate efforts to strengthen public health and support the critical importance of maintaining a smoke-free environment, especially in early life and mitigating substance abuse in adulthood.

## Supporting information

S1 Table Distribution of sleep problems in adults. (DOCX)

S1 Text PLOS questionnaire on inclusivity in global health. (DOCX)

S1 Data Dataset for adults information. (CSV)

S2 Data Dataset for children information. (CSV)

## List of abbreviations

AIC: Akaike Information Criterion
COVID-19: Coronavirus Disease
DAG: Directed Acyclic Graph
GAD: Generalized anxiety disorder
PHQ: Patient Health Questionnaire
SDB: Sleep-disordered breathing problem
SHS: Second-hand smoke
