# Supplementary material for: Association between early life second-hand smoke exposure on child sleep and psychoactive substance use on adult sleep patterns in an urban informal settlement in Uganda
Source: PLoS One. 2025 Jan 3;20(1):e0312127. doi: 10.1371/journal.pone.0312127 (PMC11698410; doi:10.1371/journal.pone.0312127)
Supplement: S1 Table — (DOCX) [file pone.0312127.s001.docx]

**S1. Table: Distribution of adult sleep problems by participant characteristics**

|  | **Total** | **Insufficient sleep** | | **Insomnia symptoms** | | **Sleep dissatisfaction** | |
| --- | --- | --- | --- | --- | --- | --- | --- |
| Attributes |  | No, n (%) | Yes, n (%) | No, n(%) | Yes, n (%) | No, n(%) | Yes, n (%) |
|  | 284 | 116 (40.9) | 168 (59.2) | 185 (65.1) | 99 (34.9) | 202 (71.1) | 82 (28.8) |
| Gender: Male |  |  |  |  |  |  |  |
| Female | 242 | 104 (43.0) | 138 (57.0) | 155(64.0%) | 87(36.0%) | 177(73.1%) | 65(26.9%) |
| Males | 42 | 12 (28.6) | 30 (71.4) | 30(71.4%) | 12(28.6%) | 25(59.5%) | 17(40.5%) |
| Age (in years) |  |  |  |  |  |  |  |
| < 30 | 146 | 65 (44.5) | 81 (55.5) | 95(65.1%) | 51(34.9%) | 102(69.9%) | 44(30.1%) |
| 30 - 45 | 108 | 41 (38.0) | 67 (62.0) | 68(63.0%) | 40(37.0%) | 80(74.1%) | 28(25.9%) |
| > 45 | 30 | 10 (33.3) | 20 (66.7) | 22(73.3%) | 8(26.7%) | 20(66.7%) | 10(33.3%) |
| Marital status |  |  |  |  |  |  |  |
| Married | 136 | 65 (47.8) | 71 (52.2) | 92(67.6%) | 44(32.4%) | 99(72.8%) | 37(27.2%) |
| Separated | 43 | 109 (20.9) | 34 (79.1) | 30(69.8%) | 13(30.2%) | 35(81.4%) | 8(18.6%) |
| Single | 105 | 42 (40.0) | 63 (60.0) | 63(60.0%) | 42(40.0%) | 68(64.8%) | 37(35.2%) |
| Occupation |  |  |  |  |  |  |  |
| Business | 137 | 55 (40.2) | 82 (59.9) | 86(62.8%) | 51(37.2%) | 89(65.0%) | 48(35.0%) |
| Employed | 58 | 21 (36.2) | 37 (63.8) | 39(67.2%) | 19(32.8%) | 42(72.4%) | 16(27.6%) |
| Unemployed | 72 | 32 (44.4) | 40 (55.6) | 11(64.7%) | 6(35.3%) | 15(88.2%) | 2(11.8%) |
| Other | 17 | 8 (47.1) | 9 (52.9) | 49(68.1%) | 23(31.9%) | 56(77.8%) | 16(22.2%) |
| Household income |  |  |  |  |  |  |  |
| < 50 | 77 | 29 (37.7) | 48 (62.3) | 57(74.0%) | 20(26.0%) | 60(77.9%) | 17(22.1%) |
| 50 – 150 | 163 | 73 (44.8) | 90 (55.2) | 105(64.4%) | 58(35.6%) | 106(65.0%) | 57(35.0%) |
| >150 | 44 | 14 (31.8) | 30 (68.2) | 23(52.3%) | 21(47.7%) | 36(81.8%) | 8(18.2%) |
| PM2.5 levels |  |  |  |  |  |  |  |
| Low | 136 | 45 (33.1) | 91 (66.9) | 90(66.2%) | 46(33.8%) | 100(73.5%) | 36(26.5%) |
| High | 148 | 71 (48.0) | 77 (52.0) | 95(64.2%) | 53(35.8%) | 102(68.9%) | 46(31.1%) |
| Carbon monoxide levels |  |  |  |  |  |  |  |
| Low | 137 | 65 (47.5) | 72 (52.5) | 84(61.3%) | 53(38.7%) | 94(68.6%) | 43(31.4%) |
| High | 147 | 51 (34.7) | 96 (65.3) | 101(68.7%) | 46(31.3%) | 108(73.5%) | 39(26.5%) |
| Current smoking |  |  |  |  |  |  |  |
| No | 243 | 103(42.4) | 140 (57.6) | 149(61.3%) | 94(38.7%) | 175(72.0%) | 68(28.0%) |
| Yes | 41 | 13 (31.7) | 28 (68.3) | 36(87.8%) | 5(12.2%) | 27(65.9%) | 14(34.1%) |
| Marijuana use |  |  |  |  |  |  |  |
| No | 265 | 109 (41.1) | 156 (58.9) | 168(63.4%) | 97(36.6%) | 193(72.8%) | 72(27.2%) |
| Yes | 19 | 7 (36.8) | 12 (63.2) | 17(89.5%) | 2(10.5%) | 9(47.4%) | 10(52.6%) |
| Alcohol use |  |  |  |  |  |  |  |
| No | 175 | 66 (37.7) | 109 (62.3) | 104(59.4%) | 71(40.6%) | 139(79.4%) | 36(20.6%) |
| Yes | 109 | 50 (45.9) | 59 (54.1) | 81(74.3%) | 28(25.7%) | 63(57.8%) | 46(42.2%) |
| Indoor spraying for insecticides |  |  |  |  |  |  |  |
| No | 196 | 79 (40.3) | 117 (59.7) | 121(61.7%) | 75(38.3%) | 146(74.5%) | 50(25.5%) |
| Yes | 88 | 37 (40.9) | 51 (58.0) | 64(72.7%) | 24(27.3%) | 56(63.6%) | 32(36.4%) |
| Hypertension |  |  |  |  |  |  |  |
| No | 202 | 84 (41.6) | 118 (58.4) | 128(63.4%) | 74(36.6%) | 150(74.3%) | 52(25.7%) |
| Yes | 80 | 32 (40.0) | 48 (60.0) | 56(70.0%) | 24(30.0%) | 50(62.5%) | 30(37.5%) |
| Have children under 5 |  |  |  |  |  |  |  |
| No | 128 | 40 (31.3) | 88 (68.8) | 83(64.8%) | 45(35.2%) | 90(70.3%) | 38(29.7%) |
| Yes | 156 | 76 (48.7) | 80 (51.3) | 102(65.4%) | 54(34.6%) | 112(71.8%) | 44(28.2%) |
| Self-reported poor health |  |  |  |  |  |  |  |
| No | 162 | 63 (38.9) | 99 (61.1) | 103(63.6%) | 59(36.4%) | 122(75.3%) | 40(24.7%) |
| Yes | 122 | 53(43.4) | 69 (59.2) | 82(67.2%) | 40(32.8%) | 80(65.6%) | 42(34.4%) |
